# Immune responses after one versus two Influenza A/B vaccinations in patients with multiple myeloma

**DOI:** 10.1007/s00277-025-06367-1

**Published:** 2025-05-15

**Authors:** Julius C. Enssle, Tonio Brinkschmidt, Ralf Dürrwald, Sebastian Wolf, David Zurmeyer, Björn Steffen, Evelyn Ullrich, Thomas Oellerich, Hubert Serve, Ivana von Metzler

**Affiliations:** 1 Department of Medicine II - Hematology and Oncology, Goethe-University, University Hospital, Frankfurt Am Main, Germany; 2 Frankfurt Cancer Institute (FCI), Frankfurt Am Main, Germany; 3 German Cancer Research Center (DKFZ), partner site Frankfurt, a partnership between German Cancer Consortium (DKTK), University Hospital Frankfurt, Frankfurt Am Main, Germany; 4 University Cancer Center (UCT), Frankfurt Am Main, Germany; 5 Influenza National Reference Center, Robert Koch Institute, Berlin, Germany; 6 Experimental Immunology, Department for Children and Adolescents Medicine, Goethe-University, University Hospital, Frankfurt Am Main, Germany; 7 Division of Pediatric Stem Cell Transplantation and Immunology, Department for Children and Adolescents Medicine, Goethe-University, University Hospital, Frankfurt Am Main, Germany

**Keywords:** Influenza A/B, Vaccination, Multiple myeloma, Serological and immune cell response

## Abstract

Seasonal Influenza A/B vaccination is routinely administered in patients with Multiple Myeloma (MM) given their disease-and therapy-associated immunosuppression and risk of infection. Previous data show poor seroconversion rates after one vaccination with an increase to ~ 60% after boosting while the impact of multiple lines of therapy remains unclear. Accordingly, we performed a retrospective single-center study assessing immune responses after single or prime-boosting vaccination in 71 patients with MM treated at our institution during the 2019/20 season. Overall, 63.3% of patients with MM achieved sufficient responses after one or two Influenza A/B vaccinations. In patients receiving a prime-boost approach, significantly higher serological titers but no significant increase in responder rates were observed after the boost vaccination. Complete or very good partial remission and no immunoparesis were identified as independent predictors of sufficient serological response by multivariate regression analysis and responders were characterized by high CD19^+^ B-cell and CD4^+^ T-cell counts. Patients achieving a sufficient response only after the prime-boost approach showed significantly shorter time since high-dose chemotherapy and autologous stem-cell transplantation (HDC-ASCT). Together, this study suggests that single vaccination against Influenza A/B might be sufficient for patients with MM while a prime-boost approach might be necessary for patients with recent HDC-ASCT.

**Supplementary Information:**

The online version contains supplementary material available at 10.1007/s00277-025-06367-1.

## Introduction

Multiple myeloma (MM) is a hematological malignancy originating from clonal plasma cells, which is associated with high levels of immunoparesis. The treatment includes diverse agents also targeting B-cells and frequently requires intensive regimens like high-dose chemotherapy followed by autologous stem cell transplantation (auto-HSCT) [1]. As a result, patients with MM are highly vulnerable to infections [2]. Respiratory infections, often associated with viruses such as Influenza A/B, are more likely to follow a severe course and represent a leading cause of death [3, 4]. Hence, protective measures through efficient vaccination strategies are of particular importance in patients with MM.

Influenza A/B infections are estimated to be responsible for approximately 5 million hospitalizations and about 290,000 to 650,000 deaths worldwide each year [5, 6]. While infection is often asymptomatic or mild in healthy individuals, morbidity and mortality are significantly increased in cancer patients [7]. Seasonal immunizations with a tetravalent vaccine against four Influenza virus strains (two A subtypes and two B subtypes) lead to improved infection-related outcome in high-risk groups [8]. Therefore, annual Influenza A/B vaccination is recommended for patients with MM and is an established component of clinical care [9, 10]. However, vaccine response is significantly reduced in MM patients and is further compromised by anti-tumor treatment [11]. Additionally, whether all MM patients require a prime-boost approach to overcome such constraints and achieve a sufficient immune response and protection is matter of debate [12–14]. So far, the impact of novel therapeutic agents—such as anti-CD38 antibodies – on the Influenza A/B immunization success has not been studied. Accordingly, we performed a retrospective single-center observational study on 71 MM patients. Our aim was to gain comprehensive insight into the immune response monitoring clinical features, serological response, and immune cell status during Influenza A/B immunization.

## Materials and methods

### Study design and participants

To study responses to Influenza A/B immunization, we initialized a retrospective observational study on 71 patients with MM, who were eligible for vaccination against Influenza A/B, at the Hematology/Oncology Department, University Hospital Frankfurt, Germany, between September 15th, 2020, and June 30th, 2021. All patients received at least one dose of tetravalent Influenza A/B vaccine (Influvac tetra 2020/2021, Mylan Healthcare GmBH, Troisdorf, Germany). Upon patient´s choice and without knowledge of response after the first dose, 52 of 71 MM patients received a second application of the tetravalent Influenza A/B vaccine, which was given at a median of 29 days (range 21–49 days) after the first shot. Vaccination was performed either at the Hematology/Oncology Department, University Hospital Frankfurt or upon patient’s choice by the primary care physician. Peripheral blood (PB) samples were obtained during routine laboratory examinations before the first vaccination (timepoint TP1), before the second (TP2) and three to four weeks after the second vaccination or 6–8 weeks after the first vaccination in case of no second vaccination (TP3). Basic disease and clinical characteristics of the patients were retrieved from the electronic health care record system at our institution. The primary endpoint was a sufficient serological response as defined by an at least four-fold increase in antibody titers or a final titer ≥ 1:40. All patients declared written informed consent. This study was approved by the local ethics committee Frankfurt, Germany (Ethics vote number: UCT-70–2020).

### Sample preparation

Patients’ whole blood was collected in Lithium Heparin tubes (Sarstedt, Germany) and Serum tubes (Sarstedt, Germany). Serological assays were performed from serum and all immune cell monitoring was performed on patients’ whole blood (see below).

### Immune monitoring analysis

Immune cell subtypes were quantified via Sysmex and flow cytometry from patients’ whole blood. Absolute cell counts of leukocytes, lymphocytes, neutrophils, monocytes and thrombocytes were derived by Sysmex analysis. Patients’ whole blood was analyzed with the BD MultitestTM 6-color TBNK and the BD MultitestTM CD8/CD38/CD3/HLA-DR staining kits (both BD Biosciences, Germany) according to the manufacturer ´s protocols. The percentage of lymphocytes, CD4 + T-cells, CD8 + T-cells, CD19 + B-cells, CD56 + CD16 + NK-cells and activated T-cells (CD8 + CD38 + and CD3 + HLA-DR + T-cells) were determined using the FACS Canto II flow cytometer and the FACS Canto software (BD Biosciences, Germany). Absolute cell counts of immune cell subtypes were calculated based on the lymphocyte count by the Sysmex analysis.

### Hemagglutinin inhibition (HAI) assay

The respective sera pre and post Influenza A/B vaccination were investigated by HAI assay. Egg-propagated vaccine viruses of the Influenza season 2020–2021 and A(H1N1) pdm09 vaccine virus of the Influenza season 2020 (southern hemisphere) were used (Tab. S5). HAI assay employed turkey red blood cells; viruses were adjusted to 8 hemagglutinating units for analysis. Positive and negative control sera were carried along by using ferret antisera against each of the vaccine viruses used and sera of not immunized ferrets.

### Statistical analysis

For statistical analysis, R version 4.1. was used (R Core Team, 2021). Continuous variables were compared with the Mann–Whitney-U test for two independent groups and Kruskal–Wallis test for three or more independent groups, categorical variables with the Fisher’s exact test and the chi-square test. P-values were adjusted by the Benjamini–Hochberg method for multiple testing. Logistic regression was calculated for multivariate analysis. For logarithmic graphical visualisation, continuous variables were transformed by addition of 1 to each value.

## Results

Overall, 71 patients with MM were analyzed in this study. The median age was 67 years, and the predominant MM subtype was IgG (*n* = 33; 46.5%) with standard risk cytogenetics (*n* = 46, 64.8%) (Tab.1). 52.1% of patients (*n* = 37) were undergoing active treatment, 26.8% of patients were maintained with a lenalidomide-based protocol and 64.8% of patients were in their first line of therapy. The majority of patients displayed at least very good complete remission (VGPR) (*n* = 49, 69.0%) and 78.9% of patients (*n* = 56) received at least one previous high-dose chemotherapy and autologous stem-cell transplantation (HDC-ASCT). 52 patients (73.2%) received a prime-boost-approach while the other 19 patients (26.8%) only received one vaccination.

**Table 1:** Patient characteristics

	Overall	Non-Respnder	Responder	*p*-value
Patients, *n* (%)	71 (100.0)	26 (36.7)	45 (63.3)	
Female sex, *n* (%)	32 (45.1)	10 (38.5)	22 (48.9)	0.546
Age, median [IQR]	67.00 [60.50, 73.00]	69.50 [66.00, 72.00]	64.00 [60.00, 73.00]	0.214
Type MM, *n* (%)				0.546
IgG	33 (46.5)	12 (46.2)	21 (46.7)	
LC	19 (26.8)	9 (34.6)	10 (22.2)	
IgA	18 (25.4)	5 (19.2)	13 (28.9)	
IgD	1 (1.4)	0 (0.0)	1 (2.2)	
revised ISS, *n* (%)				
1	25 (35.2)	11 (42.3)	14 (31.1)	
2	27 (38.0)	8 (30.8)	19 (42.2)	
3	11 (15.5)	5 (19.2)	6 (13.3)	
NA	8 (11.3)	2 (7.7)	6 (13.3)	
High-risk cytogenetics, *n* (%)				0.539
no	46 (64.8)	19 (73.1)	27 (60.0)	
yes	18 (25.4)	5 (19.2)	13 (28.9)	
NA	7 (9.9)	2 (7.7)	5 (11.1)	
Remission status, *n* (%)				0.007
CR/VGPR	49 (69.0)	12 (46.2)	37 (82.2)	
PR	11 (15.5)	6 (23.1)	5 (11.1)	
SD	3 (4.2)	3 (11.5)	0 (0.0)	
PD	8 (11.3)	5 (19.2)	3 (6.7)	
HDCT, *n* (%)				0.66
0	15 (21.1)	6 (23.1)	9 (20.0)	
1	35 (49.3)	14 (53.8)	21 (46.7)	
2	21 (29.6)	6 (23.1)	15 (33.3)	
Time since last HSCT, median [IQR]	13.00 [4.00, 32.75]	9.50 [3.75, 24.50]	23.00 [5.00, 35.25]	0.346
Current line of therapy, *n* (%)				0.097
1	46 (64.8)	12 (46.2)	34 (75.6)	
2	11 (15.5)	4 (15.4)	7 (15.6)	
3	6 (8.5)	3 (11.5)	3 (6.7)	
4	4 (5.6)	3 (11.5)	1 (2.2)	
5	1 (1.4)	1 (3.8)	0 (0.0)	
6	1 (1.4)	1 (3.8)	0 (0.0)	
7	1 (1.4)	1 (3.8)	0 (0.0)	
10	1 (1.4)	1 (3.8)	0 (0.0)	
Status of therapy, *n* (%)				0.02
no therapy	15 (21.1)	2 (7.7)	13 (28.9)	
maintenance	19 (26.8)	5 (19.2)	14 (31.1)	
on therapy	37 (52.1)	19 (73.1)	18 (40.0)	
previous PI-based, *n* (%)	68 (95.8)	25 (96.2)	43 (95.6)	1
previous IMID-based, *n* (%)	61 (85.9)	22 (84.6)	39 (86.7)	1
previous anti-CD38-based, *n* (%)	24 (33.8)	13 (50.0)	11 (24.4)	0.553
current PI-based, *n* (%)	10 (14.1)	5 (19.2)	5 (11.1)	0.553
current IMID-based, *n* (%)	33 (46.5)	13 (50.0)	20 (44.4)	0.837
current anti-CD38-based, *n* (%)	11 (15.5)	6 (23.1)	5 (11.1)	0.316
Immunoparesis, *n* (%)				< 0.001
no	29 (40.8)	3 (11.5)	26 (57.8)	
yes	40 (56.3)	21 (80.8)	19 (42.2)	
NA	2 (2.8)	2 (7.7)	0 (0.0)	
Prime/boost vaccination, *n* (%)	52 (73.2)	16 (61.5)	36 (80.0)	0.157

**Fig. 1:**
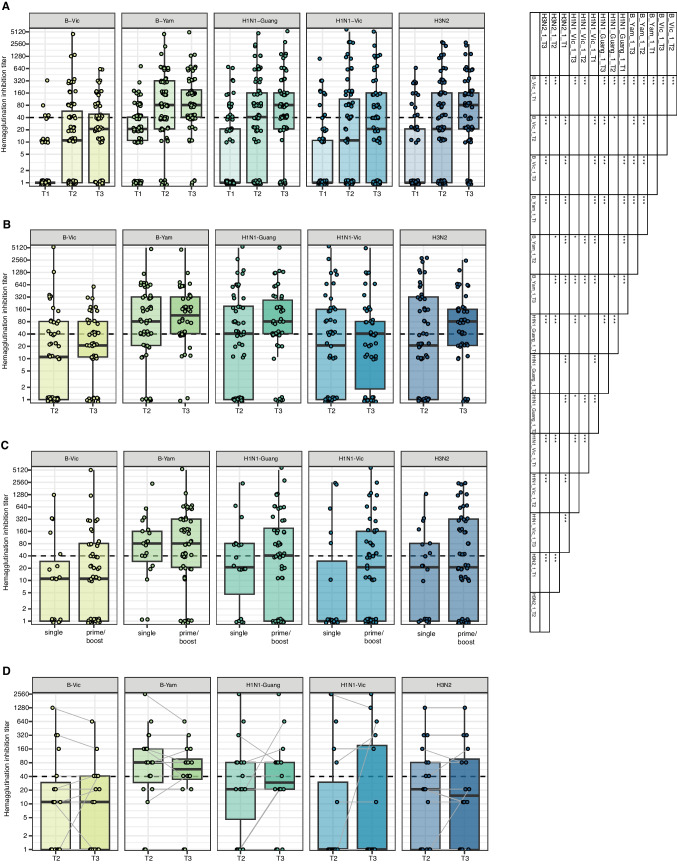
Neutralization titers after Influenza A/B vaccinations in patients with MM. **A** Hemagglutinin inhibition titers against Influenza A/B virus strains (for abbreviations see Tab.S5) measured by *Hemagglutinin inhibition (HAI) assay *in all patients with MM before the first vaccination (T1), after the first vaccination (T2) and in case of after the second vaccination or at long-term (42–56 days) timepoint (T3). Statistical testing results are depicted in the accompanying table (right side). **B** Hemagglutinin inhibition titers against Influenza A/B strains in patients who received a prime-boost vaccination approach. **C **Hemagglutinin inhibition titers after second vaccination (prime-boost approach-receiving patients) or at long-term timepoint in case of single vaccination. **D **Hemagglutinin inhibition titers after the first vaccination (T2) compared to long-term timepoint (T3) in patients with a single Influenza A/B vaccination. Dashed lines denote the protective neutralization titer of ≥ 1:40. Statistical testing in A was performed by pairwise Wilcoxon-test with multiple hypothesis correction by the Benjamini-Hochberg method. Statistical testing in B-D was performed by Wilcoxon-test. *P*-values are reported as * < 0.05, ** < 0.01, *** < 0.001

To investigate the serological response, we assessed the hemagglutinin inhibition (HI) titers before the first, 21–28 days and 42–56 days after the first vaccination (in case of second vaccination, 21–28 days afterwards) against 5 prevalent Influenza A/B strains. Here, 8 patients (11.3%) showed HI titers ≥ 1:40 before the annual vaccination. Further, we observed significant increases in HI titers already after the first vaccination (Fig. 1A). Relevant differences between the individual strains were observed with *B-Victoria* strain showing the poorest increase in HI titer levels (Fig. 1A). However, when HI titers after the first vaccination were compared to those after the second vaccination in patients with a prime-boost approach, no significant differences were observed (Fig. 1B). Also, the patients with a prime-boost vaccination showed no significant differences in HI titers at d42-d56 compared to the single vaccination group (Fig. 1C). In line, no significant waning effect was observed from post-vaccination d21-d28 to d42-d56 in patients with a single Influenza A/B vaccination (Fig. 1D).

**Fig. 2:**
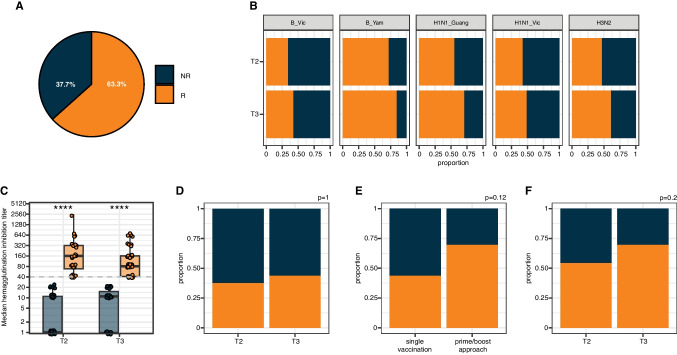
Serological responder status after Influenza A/B vaccinations in patients with MM. **A **Overall frequency of serological responders (R, orange) and non-responders (NR, blue) after one (single vaccination patients with MM) or two (prime-boost approach patients with MM) Influenza A/B vaccinations. **B** Proportion of serological (non-) responders after one (single vaccination patients with MM) or two (prime-boost approach patients with MM) Influenza A/B vaccinations stratified for the different timepoints and individual Influenza A/B strains. **C **Median hemagglutinin inhibition across all strains determined by HAI assay and stratified for serological responders (orange) and non-responders (blue). Dashed line denotes the protective neutralization titer of ≥ 1:40. **D **Proportion of serological (non-) responders after first vaccination (T2) or at long-term (T3, 42–56 days) in patients with only one immunization. **E **Proportion of vaccination (non-) responders in patients with one vaccination compared to patients receiving a prime-boost approach. **F** Proportion of vaccination (non-) responders after first (T2) or second (T3) vaccination in patients with a prime-boost approach. Statistical testing in C was performed by Wilcoxon-test with **** *p*-value < 0.0001. Statistical testing in B,D-F was performed by Chi-Square-test

Next, we assessed the frequency of a sufficient serological response defined as a median HI titer across all 5 strains of ≥ 1:40 or a four-fold increase in HI titers after the vaccination event. In general, 63.3% of patients (*n* = 45) achieved a sufficient serological response status after one or two vaccinations (Fig. 2A, Tab. 1). Similar frequencies were observed for each individual strain (Fig. 2B). When analyzed for the median HI titer, responders and non-responders significantly separated at both post-vaccination timepoints (Fig. 2C). Importantly, patients achieving a sufficient serological response after the first vaccination and not receiving a second vaccination did maintain this response at d42-d56 (Fig. 2D). Although trending towards higher sufficient responder rates, no significant differences were observed when patients with prime-boost approach were compared to those receiving a single vaccination (Fig. 2E) or when the responder frequencies were compared after the first or second vaccination in those receiving a prime-boost approach (Fig. 2F). Regarding disease- and clinical characteristics, responders showed significantly higher rates of CR/VGPR, were more frequently without current therapy but within current maintenance therapy and displayed significantly lower rates of immunoparesis (Tab.1). Regarding individual substances, responders trended towards lower previous exposure to anti-CD38-including treatment regiments (*p* = 0.053), while no significant differences for imid-based, proteasome-inhibition (PI)-based previous or current therapy were observed (Tab.1). While the rate of sufficient responder was lower if only assessed after one vaccination compared to all patients receiving a prime-boost approach, these differences were not statistically significant (Fig. 2E**, **Tab. S1-S2). The differences in the clinical characteristics of vaccination responders remained similar to the above-mentioned patterns when the stratification into (non-) responders was performed after the first vaccination and for all patients with prime-boosting (Tab. S1-S2). Interestingly, when dichotomized after the first vaccination, non-responders showed significantly lower duration since the last HDC-ASCT (Tab. S1). Accordingly, when we compared early responders (achieving sufficient response after the first vaccination) to late responders (sufficient response only after second vaccination), late responder also displayed significantly shorter duration since the last HDC-ASCT (Tab. S3).

**Fig. 3:**
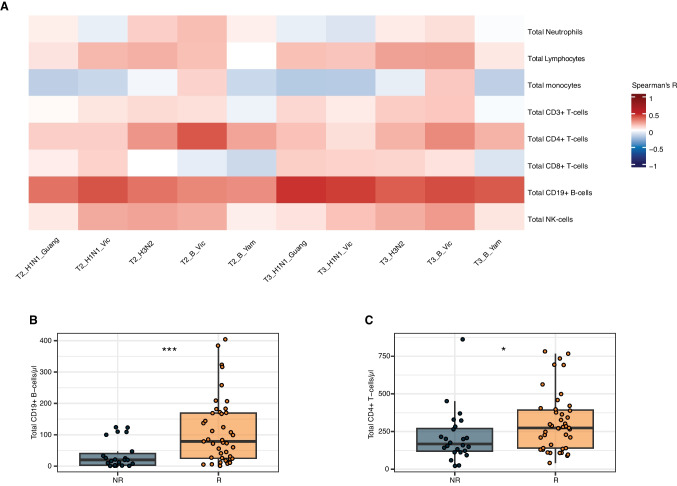
Peripheral immune cell patterns in patients with MM receiving Influenza A/B vaccination. **A** Pairwise Spearman-correlation analysis of neutralization titers for indicated Influenza A/B strains (for abbreviations see Tab. S5) after first (T2) and second Influenza A/B vaccination or long-term timepoint (T3) with determined peripheral immune cell counts before the first Influenza A/B vaccination. **B **Peripheral CD19^+^ B-cell counts stratified for serological response status (R, response; NR, non-response). **C **Peripheral CD4^+^ T-cell counts stratified for serological response status (R, response; NR, non-response). Statistical testing in B-C was performed by Wilcoxon-test. *P*-values are reported as * < 0.05 and *** < 0.001

To evaluate the correlation of the patient’s cellular immune system with the serological response after one or two Influenza A/B vaccinations, we measured the peripheral immune cell populations before the first vaccination. Here, there was a strong positive correlation between CD19^+^ B-cell counts with the HI titers across all strains and timepoints (Fig. 3A). This was also observed to a lesser extent for HI titers and CD4^+^ T-cell and NK-cell levels (Fig. 3A). Stratifying again by achievement of a sufficient serological response, vaccination responders displayed significantly higher CD19^+^ B-cell and CD4^+^ T-cell counts (Fig. 3B-C, Tab. S4).

To test whether one of the observed clinical factors and immune cell counts were different between (non-) responders, we fitted a multivariate logistic regression model for achievement of sufficient response as defined above (Tab.2). Only absent immunoparesis (OR 5.37 [95% CI 1.13–33.21], *p* = 0.046) and present CR/VGPR (OR 4.07 [95% CI 1.09–16.57], *p* = 0.04) were identified as independent predictors for sufficient serological response with CD19^+^ B-cell counts and current active therapy as included covariates.

**Table 2:** Multivariate logistic regression for serologic response status

	OR	2.50%	97.50%	*p*.value
CD19+ B-cell counts	1.00888872	0.99905689	1.02180345	0.11898708
On therapy	1.10939978	0.25841577	5.13994552	0.8899939
No immunoparesis	5.36604661	1.13113827	33.210019	0.045843
CR/VGPR	4.075291	1.0873796	16.572314	0.04008177

## Discussion

The current study highlights the effectiveness of the tetravalent Influenza A/B vaccine in multiple myeloma (MM) patients, with 63.3% of the patients achieving a sufficient serological response status based on antibody level monitoring. To define the serological response, we used HI titers, which are widely recognized as predominant serological correlates of vaccination response [15]. However, titers should only be viewed as surrogate markers for vaccine responsiveness, as the clinical benefit of a titer of ≥ 1:40 has only been confirmed for healthy young individuals, not for MM patients [12, 16]. Given the limited size of the patient cohort and the retrospective nature of the study, additional clinical aspects, such as potential previous respiratory infections, could not be comprehensively investigated, although they can significantly influence vaccine responsiveness and pre-existing immunity [13].

The analysis of the entire patient cohort revealed a significant increase in neutralizing titers against each examined strain after the first vaccination. The results exceeded responder rates reported in previous studies on vaccine response in MM patients or patients with other hematological disorders, probably related to the high frequency of patients in CR/VGPR [13, 14, 17]. Unlike in those studies, the overall patient cohort did not benefit from a second vaccination. General responder rates did not increase significantly after the second vaccination, and there was no pronounced waning within the follow up period of 4 weeks after only one vaccination. So far, current recommendations include to generally vaccinate twice against Influenza A/B in MM patients if there is no opportunity for antibody testing after the first vaccination [10, 12]. However, a general double vaccination is not mandatory in light of the here presented data.

Since our data does not support the general double-immunization approach, we sought to identify those patients who would benefit from a second vaccination. We highlighted several clinical characteristics as risk factors for poor vaccination response. While trends towards higher fractions of responders were observed for patients in their first line of therapy, this cannot be manifested with statistical significance. However, and in line with Branagan et al., responders were in a better remission status at the time of vaccination, showed no immunoparesis and were not undergoing classical chemotherapy beyond maintenance therapy [14]. Previous exposure to anti-CD38-including treatment regiments was another risk factor for reduced serologic response to vaccination. The inhibitory effect on the immune response associated with a targeted depletion of B cells, and an interruption of antibody formation was recently also observed in connection with the COVID- 19 vaccination [18, 19]. However, no patients with recent treatment by T-cell redirecting bispecific antibodies or CAR-T cells targeting BCMA were included in the study. Importantly, patients failing to achieve a sufficient response after the first vaccination showed significantly shorter time since their last HDC-ASCT (median time 8.0 months). In addition, stratifying by early/late vaccination response (responder state after one/after two vaccinations) revealed that especially patients who had recently received HDC-ASCT (median time since last HDC-ASCT 5.0 months) achieved responder status only after the second vaccination. In line with these findings, Hahn et al. have observed that there is an inverse correlation between the time after transplant and the increase in vaccination titers [13]. Furthermore, it has been shown that HDC-ASCT induces significant and extended immunosuppression, affecting the immune system's ability to adequately respond to vaccinations [20]. Together, this supports a favorable immunization outcome in MM patients with recent HDC-ASCT by a prime and boost approach.

To gain a more comprehensive understanding of the immune response after vaccination, we supplemented the titer test with monitoring the cellular immune response. We observed the achievement of a serological responder status to correlate with the abundance of CD19^+^ B cells, CD4^+^ T cells, and NK cells. In line with this, non-responders were characterized by a reduced frequency of these cell types. We previously detected similar differences in vaccination response related to cell composition in connection with the SARS-CoV-2 vaccination in MM patients [21–23]. Preliminary reports indicate that MM disease with a high tumor burden and therapy with B-cell depleting substances and IMIDs result in similar cellular changes regarding T and NK cells [24]. These specific changes are amplified in situations of uncontrolled disease and following HDC-ASCT [25]. In regard to anti-SARS-CoV-2 vaccination, low CD4 + T-cell proportions aligned with reduced serological response, but functional T-cell vaccination response remained non-correlated with serological vaccination [21, 23]. Hence, it is of high relevance to investigate the functional T-cell response beyond the present investigation at granular detail.

In summary, through our comprehensive analysis of cellular and serologic immune responses of MM patients to Influenza A/B vaccination, we were able to demonstrate the advantages of seasonal Influenza A/B vaccination and to define a group of MM patients who benefit from a prime- and booster vaccination. Based on our data, it is advisable to administer two vaccinations to those patients who have recently received HDC-ASCT and have a variety of risk factors for poor vaccine response. However, the systematic examination of double vaccination warrants attention. A more extensive and stratified patient cohort is essential for a thorough evaluation of the relationship between immunization outcomes and clinical factors as well as detailed evaluation of cellular responses. Additionally, it is crucial to investigate clinical effectiveness, especially regarding its influence on Influenza A/B infection, morbidity, and mortality.

## Supplementary Information

Below is the link to the electronic supplementary material.

Supplementary file1 (PDF 206 KB)

## Data Availability

Any information required to re-analyze the data reported in this paper is available from the lead contact upon request.

## References

[1] Rajkumar SV (2022) Multiple myeloma: 2022 update on diagnosis, risk stratification, and management. Am J Hematol 97:1086–1107. 10.1002/AJH.26590 35560063 10.1002/ajh.26590 PMC9387011

[2] Karlsson J, Andréasson B, Kondori N et al (2011) Comparative study of immune status to infectious agents in elderly patients with multiple myeloma, Waldenstrom’s macroglobulinemia, and monoclonal gammopathy of undetermined significance. Clin Vaccine Immunol 18:969–977. 10.1128/CVI.00021-11 21508164 10.1128/CVI.00021-11 PMC3122605

[3] Augustson BM, Begum G, Dunn JA et al (2005) Early mortality after diagnosis of multiple myeloma: analysis of patients entered onto the United kingdom Medical Research Council trials between 1980 and 2002–Medical Research Council Adult Leukaemia Working Party. J Clin Oncol 23:9219–9226. 10.1200/JCO.2005.03.2086 16275935 10.1200/JCO.2005.03.2086

[4] Brioli A, Klaus M, Sayer H et al (2019) The risk of infections in multiple myeloma before and after the advent of novel agents: a 12-year survey. Ann Hematol 98:713–722. 10.1007/S00277-019-03621-1 30680505 10.1007/s00277-019-03621-1

[5] Iuliano AD, Roguski KM, Chang HH et al (2018) Estimates of global seasonal influenza-associated respiratory mortality: a modelling study. Lancet 391:1285–1300. 10.1016/S0140-6736(17)33293-2 29248255 10.1016/S0140-6736(17)33293-2 PMC5935243

[6] Lafond KE, Porter RM, Whaley MJ et al (2021) Global burden of influenza-associated lower respiratory tract infections and hospitalizations among adults: A systematic review and meta-analysis. PLoS Med 18:. 10.1371/JOURNAL.PMED.1003550 10.1371/journal.pmed.1003550 PMC7959367 33647033

[7] Uyeki TM, Hui DS, Zambon M et al (2022) Influenza Lancet 400:693–706. 10.1016/S0140-6736(22)00982-5 36030813 10.1016/S0140-6736(22)00982-5 PMC9411419

[8] Bitterman R, Eliakim-Raz N, Vinograd I et al (2018) Influenza vaccines in immunosuppressed adults with cancer. Cochrane Database Syst Rev 2:. 10.1002/14651858.CD008983.PUB3 10.1002/14651858.CD008983.pub3 PMC6491273 29388675

[9] Ludwig H, Boccadoro M, Moreau P et al (2021) Recommendations for vaccination in multiple myeloma: a consensus of the European Myeloma Network. Leukemia 35:31–44. 10.1038/S41375-020-01016-0 32814840 10.1038/s41375-020-01016-0 PMC7787974

[10] Raje NS, Anaissie E, Kumar SK et al (2022) Consensus guidelines and recommendations for infection prevention in multiple myeloma: a report from the International Myeloma Working Group. Lancet Haematol 9:e143–e161. 10.1016/S2352-3026(21)00283-0 35114152 10.1016/S2352-3026(21)00283-0

[11] Barrett AJJ (2011) Fighting the flu in multiple myeloma. Blood 117:1–2. 10.1182/BLOOD-2010-10-311217 21212286 10.1182/blood-2010-10-311217

[12] Ludwig H, Kumar S (2023) Prevention of infections including vaccination strategies in multiple myeloma. Am J Hematol 98(Suppl 2):S46–S62. 10.1002/AJH.26766 36251367 10.1002/ajh.26766

[13] Hahn M, Schnitzler P, Schweiger B et al (2015) Efficacy of single versus boost vaccination against influenza virus in patients with multiple myeloma. Haematologica 100:e285–e288. 10.3324/HAEMATOL.2014.116772 25820335 10.3324/haematol.2014.116772 PMC4486243

[14] Branagan AR, Duffy E, Albrecht RA et al (2017) Clinical and Serologic Responses After a Two-dose Series of High-dose Influenza Vaccine in Plasma Cell Disorders: A Prospective, Single-arm Trial. Clin Lymphoma Myeloma Leuk 17:296-304.e2. 10.1016/J.CLML.2017.02.025 28343904 10.1016/j.clml.2017.02.025 PMC5413398

[15] Brydak LB, Roszkowska-Blaim M, MacHala M et al (2000) Antibody response to influenza immunization in two consecutive epidemic seasons in patients with renal diseases. Vaccine 18:3280–3286. 10.1016/S0264-410X(00)00126-2 10869773 10.1016/s0264-410x(00)00126-2

[16] Hannoun C, Megas F, Piercy J (2004) Immunogenicity and protective efficacy of influenza vaccination. Virus Res 103:133–138. 10.1016/j.virusres.2004.02.025 15163501 10.1016/j.virusres.2004.02.025

[17] Ljungman P, Engelhard D, de la Cámara R et al (2005) Vaccination of stem cell transplant recipients: recommendations of the Infectious Diseases Working Party of the EBMT. Bone Marrow Transplant 35:737–746. 10.1038/SJ.BMT.1704870 15750612 10.1038/sj.bmt.1704870

[18] Terpos E, Gavriatopoulou M, Ntanasis-Stathopoulos I et al (2021) The neutralizing antibody response post COVID-19 vaccination in patients with myeloma is highly dependent on the type of anti-myeloma treatment. Blood Cancer J 11:. 10.1038/S41408-021-00530-3 10.1038/s41408-021-00530-3 PMC8327056 34341335

[19] Terao T, Naduka T, Ikeda D et al (2022) Depletion of CD38-positive regulatory T cells by anti-CD38 monoclonal antibodies induces a durable response to SARS-CoV-2 vaccination in patients with plasma cell dyscrasia. Br J Haematol 197:417–421. 10.1111/BJH.18079 35172374 10.1111/bjh.18079 PMC9111412

[20] Parmar H, Gertz M, Anderson EI et al (2021) Microenvironment immune reconstitution patterns correlate with outcomes after autologous transplant in multiple myeloma. Blood Adv 5:1797–1804. 10.1182/BLOODADVANCES.2020003857 33787859 10.1182/bloodadvances.2020003857 PMC8045512

[21] Enssle JC, Campe J, Büchel S et al (2022) Enhanced but variant-dependent serological and cellular immune responses to third-dose BNT162b2 vaccination in patients with multiple myeloma. Cancer Cell 40:587–589. 10.1016/J.CCELL.2022.05.003 35588736 10.1016/j.ccell.2022.05.003 PMC9116569

[22] Enssle JC, Campe J, Moter A et al (2024) Cytokine-responsive T- and NK-cells portray SARS-CoV-2 vaccine-responders and infection in multiple myeloma patients. Leukemia 38:. 10.1038/S41375-023-02070-0 10.1038/s41375-023-02070-0 PMC10776400 38049509

[23] Enßle JC, Campe J, Schwenger A et al (2022) Severe impairment of T-cell responses to BNT162b2 immunization in patients with multiple myeloma. Blood 139:137–142. 10.1182/BLOOD.2021013429 34657156 10.1182/blood.2021013429 PMC8734828

[24] Dhodapkar MV (2023) The immune system in multiple myeloma and precursor states: Lessons and implications for immunotherapy and interception. Am J Hematol 98:S4–S12. 10.1002/AJH.26752 36194782 10.1002/ajh.26752 PMC9918687

[25] Coffey DG, Maura F, Gonzalez-Kozlova E et al (2023) Immunophenotypic correlates of sustained MRD negativity in patients with multiple myeloma. Nat Commun 14:1 14:1–12. 10.1038/s41467-023-40966-8 10.1038/s41467-023-40966-8 PMC10475030 37660077

