# Supplementary material for: Immune responses after one versus two Influenza A/B vaccinations in patients with multiple myeloma
Source: Ann Hematol. 2025 May 15;104(5):2813–21. doi: 10.1007/s00277-025-06367-1 (PMC12141375; doi:10.1007/s00277-025-06367-1)
Supplement: Supplementary file 1 — Supplementary file1 (PDF 206 KB) [file 277_2025_6367_MOESM1_ESM.pdf]

Supplemental Information

Immune responses after one versus two Influenza A/B vaccinations in patients with Multiple Myeloma

Julius C. Enßle et al.

Supplemental Tables

Table S1. Patient characteristics after first vaccination

	Overall	Non-Respnder	Responder	p-value
Patients, n (%)	71	35 (49.3)	36 (50.7)	
Female sex, n (%)	32 (45.1)	14 (40.0)	18 (50.0)	0.543
Age, median [IQR]	67.00 [60.50, 73.00]	68.00 [61.50, 72.00]	64.00 [60.00, 73.00]	0.738
Type MM, n (%)				0.359
IgG	33 (46.5)	16 (45.7)	17 (47.2)	
LC	19 (26.8)	12 (34.3)	7 (19.4)	
IgA	18 (25.4)	7 (20.0)	11 (30.6)	
IgD	1 (1.4)	0 (0.0)	1 (2.8)	
revised ISS, n (%)				0.672
1	25 (35.2)	13 (37.1)	12 (33.3)	
2	27 (38.0)	11 (31.4)	16 (44.4)	
3	11 (15.5)	6 (17.1)	5 (13.9)	
NA	8 (11.3)	5 (14.3)	3 (8.3)	
High-risk cytogenetics, n (%)				0.898
no	46 (64.8)	22 (62.9)	24 (66.7)	
yes	18 (25.4)	9 (25.7)	9 (25.0)	
NA	7 (9.9)	4 (11.4)	3 (8.3)	
Remission status, n (%)				0.205
CR/VGPR	49 (69.0)	21 (60.0)	28 (77.8)	
PR	11 (15.5)	6 (17.1)	5 (13.9)	
SD	3 (4.2)	3 (8.6)	0 (0.0)	
PD	8 (11.3)	5 (14.3)	3 (8.3)	
HDCT, n (%)				0.691
0	15 (21.1)	7 (20.0)	8 (22.2)	
1	35 (49.3)	19 (54.3)	16 (44.4)	
2	21 (29.6)	9 (25.7)	12 (33.3)	
Time since last HSCT, median [IQR]	13.00 [4.00, 32.75]	8.00 [2.00, 23.50]	24.50 [9.75, 40.25]	0.02
Current line of therapy, n (%)				0.221
1	46 (64.8)	19 (54.3)	27 (75.0)	
2	11 (15.5)	5 (14.3)	6 (16.7)	
3	6 (8.5)	3 (8.6)	3 (8.3)	
4	4 (5.6)	4 (11.4)	0 (0.0)	
5	1 (1.4)	1 (2.9)	0 (0.0)	
6	1 (1.4)	1 (2.9)	0 (0.0)	
7	1 (1.4)	1 (2.9)	0 (0.0)	
10	1 (1.4)	1 (2.9)	0 (0.0)	
Status of therapy, n (%)				0.01
no therapy	15 (21.1)	3 (8.6)	12 (33.3)	
maintenance	19 (26.8)	8 (22.9)	11 (30.6)	
on therapy	37 (52.1)	24 (68.6)	13 (36.1)	
previous PI-based, n (%)	68 (95.8)	33 (94.3)	35 (97.2)	0.98
previous IMID-based, n (%)	61 (85.9)	29 (82.9)	32 (88.9)	0.697
previous anti-CD38-based, n (%)	24 (33.8)	15 (42.9)	9 (25.0)	0.18
current PI-based, n (%)	10 (14.1)	5 (14.3)	5 (13.9)	1
current IMID-based, n (%)	33 (46.5)	17 (48.6)	16 (44.4)	0.912
current anti-CD38-based, n (%)	11 (15.5)	7 (20.0)	4 (11.1)	0.48
Immunoparesis, n (%)				0.001
no	29 (40.8)	7 (20.0)	22 (61.1)	
yes	40 (56.3)	26 (74.3)	14 (38.9)	
NA	2 (2.8)	2 (5.7)	0 (0.0)	
Prime/boost vaccination, n (%)	52 (73.2)	24 (68.6)	28 (77.8)	0.543

Table S2. Patient characteristics after second vaccination

	Overall	Non-Respnder	Responder	p-value
Patients, n (%)	62	23 (37.1)	39 (62.9)	
Female sex, n (%)	26 (41.9)	8 (34.8)	18 (46.2)	0.542
Age, median [IQR]	66.50 [61.25, 72.75]	70.00 [66.00, 72.00]	64.00 [59.50, 73.00]	0.149
Type MM, n (%)				0.6
IgG	30 (48.4)	11 (47.8)	19 (48.7)	
IgA	17 (27.4)	5 (21.7)	12 (30.8)	
LC	15 (24.2)	7 (30.4)	8 (20.5)	
revised ISS, n (%)				0.55
1	19 (30.6)	8 (34.8)	11 (28.2)	
2	25 (40.3)	9 (39.1)	16 (41.0)	
3	11 (17.7)	5 (21.7)	6 (15.4)	
NA	7 (11.3)	1 (4.3)	6 (15.4)	
High-risk cytogenetics, n (%)				0.846
no	38 (61.3)	15 (65.2)	23 (59.0)	
yes	17 (27.4)	6 (26.1)	11 (28.2)	
NA	7 (11.3)	2 (8.7)	5 (12.8)	
Remission status, n (%)				0.001
CR/VGPR	43 (69.4)	9 (39.1)	34 (87.2)	
PR	9 (14.5)	6 (26.1)	3 (7.7)	
SD	3 (4.8)	3 (13.0)	0 (0.0)	
PD	7 (11.3)	5 (21.7)	2 (5.1)	
HDCT, n (%)				0.672
0	11 (17.7)	5 (21.7)	6 (15.4)	
1	31 (50.0)	12 (52.2)	19 (48.7)	
2	20 (32.3)	6 (26.1)	14 (35.9)	
Time since last HSCT, median [IQR]	13.00 [4.00, 32.00]	11.50 [4.00, 27.50]	15.00 [5.00, 32.00]	0.722
Current line of therapy, n (%)				0.115
1	38 (61.3)	9 (39.1)	29 (74.4)	
2	11 (17.7)	5 (21.7)	6 (15.4)	
3	6 (9.7)	3 (13.0)	3 (7.7)	
4	3 (4.8)	2 (8.7)	1 (2.6)	
5	1 (1.6)	1 (4.3)	0 (0.0)	
6	1 (1.6)	1 (4.3)	0 (0.0)	
7	1 (1.6)	1 (4.3)	0 (0.0)	
10	1 (1.6)	1 (4.3)	0 (0.0)	
Status of therapy, n (%)				0.04
no therapy	12 (19.4)	2 (8.7)	10 (25.6)	
maintenance	17 (27.4)	4 (17.4)	13 (33.3)	
on therapy	33 (53.2)	17 (73.9)	16 (41.0)	
previous PI-based, n (%)	59 (95.2)	22 (95.7)	37 (94.9)	1
previous IMID-based, n (%)	54 (87.1)	19 (82.6)	35 (89.7)	0.676
previous anti-CD38-based, n (%)	22 (35.5)	12 (52.2)	10 (25.6)	0.067
current PI-based, n (%)	9 (14.5)	6 (26.1)	3 (7.7)	0.107
current IMID-based, n (%)	29 (46.8)	11 (47.8)	18 (46.2)	1
current anti-CD38-based, n (%)	11 (17.7)	7 (30.4)	4 (10.3)	0.096
Immunoparesis, n (%)				0.004
no	24 (38.7)	3 (13.0)	21 (53.8)	
yes	37 (59.7)	19 (82.6)	18 (46.2)	
NA	1 (1.6)	1 (4.3)	0 (0.0)	
Prime/boost vaccination, n (%)	46 (74.2)	14 (60.9)	32 (82.1)	0.123

Table S3. Early (post 1st) versus late (post 2nd) responder in all patients with two vaccinations

	All responder	Early responder	Late responder	p-value
Patients, n (%)	33	25	8	
Female sex, n (%)	15 (45.5)	12 (48.0)	3 (37.5)	0.911
Age, median [IQR]	64.00 [56.00, 73.00]	64.00 [60.00, 74.00]	59.50 [52.50, 70.00]	0.377
Type MM, n (%)				0.383
IgG	15 (45.5)	11 (44.0)	4 (50.0)	
IgA	10 (30.3)	9 (36.0)	1 (12.5)	
LC	8 (24.2)	5 (20.0)	3 (37.5)	
revised ISS, n (%)				0.361
1	9 (27.3)	8 (32.0)	1 (12.5)	
2	12 (36.4)	9 (36.0)	3 (37.5)	
3	6 (18.2)	5 (20.0)	1 (12.5)	
NA	6 (18.2)	3 (12.0)	3 (37.5)	
High-risk cytogenetics, n (%)				0.156
no	18 (54.5)	16 (64.0)	2 (25.0)	
yes	10 (30.3)	6 (24.0)	4 (50.0)	
NA	5 (15.2)	3 (12.0)	2 (25.0)	
Remission status, n (%)				0.309
CR/VGPR	27 (81.8)	19 (76.0)	8 (100.0)	
PR	4 (12.1)	4 (16.0)	0 (0.0)	
PD	2 (6.1)	2 (8.0)	0 (0.0)	
HDCT, n (%)				0.887
0	6 (18.2)	5 (20.0)	1 (12.5)	
1	15 (45.5)	11 (44.0)	4 (50.0)	
2	12 (36.4)	9 (36.0)	3 (37.5)	
Time since last HSCT, median [IQI	24.00 [6.00, 35.50]	26.50 [9.75, 42.50]	5.00 [2.00, 17.00]	0.046
Status of therapy, n (%)				0.548
no therapy	9 (27.3)	8 (32.0)	1 (12.5)	
maintenance	11 (33.3)	8 (32.0)	3 (37.5)	
on therapy	13 (39.4)	9 (36.0)	4 (50.0)	
previous PI-based, n (%)	31 (93.9)	24 (96.0)	7 (87.5)	0.979
previous IMID-based, n (%)	29 (87.9)	23 (92.0)	6 (75.0)	0.509
previous anti-CD38-based, n (%)	8 (24.2)	7 (28.0)	1 (12.5)	0.677
current PI-based, n (%)	2 (6.1)	2 (8.0)	0 (0.0)	1
current IMID-based, n (%)	15 (45.5)	11 (44.0)	4 (50.0)	1
current anti-CD38-based, n (%)	5 (15.2)	4 (16.0)	1 (12.5)	1

Table S4. Peripheral immune cells stratified per responder status

	Overall	Non-Respnder	Responder	p-value
Patients, n (%)	67 (100.0)	24 (35.8)	43 (64.2)	
Neutrophils/ml, median [IQR]	2.36 [1.65, 3.22]	2.51 [1.69, 2.98]	2.34 [1.65, 3.87]	0.932
Lymphocytes/ml, median [IQR]	0.90 [0.58, 1.53]	0.71 [0.44, 1.46]	1.12 [0.66, 1.53]	0.209
Monocytes/ml, median [IQR]	0.49 [0.36, 0.69]	0.54 [0.32, 0.73]	0.48 [0.37, 0.66]	0.937
CD3+ T-cells/µl, median [IQR]	720.00 [368.00, 1146.00]	564.50 [274.25, 1259.50]	734.00 [380.00, 1007.50]	0.642
CD4+ T-cells/µl, median [IQR]	229.00 [134.00, 353.50]	167.50 [119.50, 269.50]	274.00 [140.00, 393.00]	0.033
CD8+ T-cells/µl, median [IQR]	389.00 [151.50, 730.00]	314.50 [110.50, 1064.25]	401.00 [225.00, 586.00]	0.794
CD19+ B-cells/µl, median [IQR]	46.00 [13.00, 123.75]	20.00 [3.00, 40.00]	79.00 [25.00, 169.50]	0.001
NK-cells/µl, median [IQR]	162.50 [64.25, 258.25]	134.00 [19.00, 262.00]	185.00 [93.50, 256.50]	0.125

Table S5. Viruses and antisera used in HAI

Vaccine virus	Titer of corresponding ferret antiserum (reciprocal)	Abbreviation
A/Guangdong-Maonan/SWL1536/2019 A(H1N1)pdm09	1280	H1N1_Guang
A/Victoria/2570/2019 A(H1N1)pdm09 HA N156K	2560	H1N1_Vic
A/Hong Kong/2671/2019 A(H3N2)	320	H3N3
B/Washington/2/2019 (B-Victoria)	80	B_Vic
B/Phuket/3073/2013 (B-Yamagata)	1280	B_Yam
Negative control	<10	
